# Monocarboxyoctyl phthalate is associated with platelet count: evidence from a large cross-sectional study

**DOI:** 10.3389/fpubh.2025.1559808

**Published:** 2025-04-25

**Authors:** Jian Zhang, Yuhan Xie, Jinqiu Chen, Lei Song

**Affiliations:** ^1^Department of Hematology, People's Hospital of Rizhao, Rizhao, China; ^2^First Clinical Medical College, Shandong University of Traditional Chinese Medicine, Jinan, China

**Keywords:** phthalate, platelet, NHANES, cross-sectional study, monocarboxyoctyl phthalate

## Abstract

**Introduction:**

Phthalates are environmental pollutants that are harmful to human health. However, the impact of phthalate on platelet count remains unclear. This study aimed to examine the correlation between five phthalate metabolites in urine and platelet count, as well as the impact of phthalate metabolite exposure on platelet count in adults.

**Methods:**

This cross-sectional study included 11,409 non-pregnant participants aged >20 years using data available from the National Health and Nutrition Examination Survey (NHANES) between 2005 and 2018. Weighted logistic regression, restricted cubic spline (RCS) modeling, and weighted quantile sum (WQS) were employed to investigate the effects of mono-(carboxyisononyl) phthalate (MCNP), mono-(carboxyoctyl) phthalate (MCOP), mono-(3-carboxypropyl) phthalate (MCPP), mono-isobutyl phthalate (MiBP) and mono-isononyl phthalate (MNP) on platelet count.

**Results:**

Logistic regression analysis suggested that MCOP [odds ratio (OR) (95% confidence interval CI) = 0.009 (0.002–0.036)] was significantly associated with the platelet count. Subgroup analysis showed negative correlations between MCOP and platelet count across all age and sex groups, and MCNP [OR (95% CI) = 0.083(0.013–0.552)] displayed a negative association with platelet count in females. MCOP had a nonlinear relationship with the platelet count in the RCS model. WQS also revealed that MCOP was related to platelet count.

**Conclusion:**

Higher urinary MCOP level was associated with lower platelet count. Further investigation is necessary to substantiate these findings, considering the shortcomings of the NHANES study.

## 1 Introduction

Phthalic acid esters (PAEs), or phthalates, are synthetic chemicals that are widely distributed in the environment and serve not only as plasticizers in industrial materials and food but also as solvents and fixatives in medical devices and personal products (1, 2). Owing to their non-covalent bonding with these products, phthalates can be easily released from the carrier into the environment under the influence of factors such as temperature and pH, and then enter the human body through dermal or dietary exposure (3, 4). In humans, phthalates are predominantly metabolized in the liver, where they are initially converted into the primary metabolite monoethyl phthalate, subsequently metabolized into phenylalanine and acetyl-CoA. In addition to the liver, phthalates may also be metabolized in the intestines, kidneys, spleen, and other organs via hydroxylation, acylation, sulfation, and glucuronidation. These metabolic processes convert phthalates into metabolites such as monomethyl phthalate, mono-hydroxybutyl phthalate, and others (5). Phthalates can be filtered by the kidneys and enter the urine. Due to the rapid metabolism and short half-life (< 24 h) of phthalates in urine, as well as the lack of active esterases that can degrade parent phthalate metabolites, urine is regarded as a suitable matrix for exposure assessment (6).

In recent years, phthalates have become one of the most prevalent environmental pollutants worldwide due to rising commercial demand and increased production (7). Growing evidence link phthalate exposure to various diseases, prompting increasingly strict regulations. The European Union adopted Directive 2005/84/EC as early as 2005, prohibiting the use of di(2-ethylhexyl) phthalate (DEHP), dibutyl phthalate, and benzyl butyl phthalate (BBP) in toys and childcare products while establishing concentration limits for other phthalates (8). In 2002, the U.S. Food and Drug Administration revised food additive regulations by removing 25 previously authorized phthalates (9). In 2024, California enacted the Toxic-Free Medical Devices Act (AB 2300), which bans the manufacture, sale, and distribution of medical devices containing specific plasticizers (10). However, the widespread use and persistence of phthalates make it a significant challenge to completely eliminate their effects. Therefore, continued regulatory and research are crucial to develop more effective strategies for mitigating phthalate exposure and its risks.

DEHP, di-isononyl phthalate (DINP), and di-isodecyl phthalate (DIDP) are authorized for use in plastic food contact materials (11). European authorities conducted a comprehensive risk assessment of DINP and DIDP, concluding that evidence did not support their classification as hazardous substances. As a result, DINP and DIDP have been regarded as alternatives to DEHP in recent years due to their lower toxicity (12, 13). Dioctyl phthalate (DnOP), an isomer of DEHP, constitutes 20% of the C6-10 phthalate mixture and is commonly used in plastics, cosmetics, floor tiles, and pesticides (14). Di-isobutyl phthalate (DiBP) is another widely utilized plasticizer, with data from the National Health and Nutrition Examination Survey (NHANES) showing a rapid increase in exposure in recent years.

The current study is based on the exposure of these five commonly used phthalates mentioned above. Mono-isononyl phthalate (MNP) and mono-(carboxyoctyl) phthalate (MCOP) are urinary metabolites of DINP. The former exhibits antiandrogenic activity and is associated with reduced sperm concentrations and counts (15), while the latter has been shown to affect thyroid function and is linked to autoimmune rheumatic diseases (16). Mono-(carboxyisononyl) phthalate (MCNP), a urinary metabolite of DIDP, has been shown to be related to the development of asthma (17). Additionally, mono-(3-carboxypropyl) phthalate (MCPP), derived from DnOP, is positively correlated with IL-6 levels and negatively correlated with serum bilirubin concentrations (18). Mono-isobutyl phthalate (MiBP), a metabolite of DiBP, is not only associated with the occurrence of diabetes and hyperuricemia but also with elevated C-reactive protein (CRP) levels, a marker of systemic inflammation (19).

Platelets, a significant component of blood, are nucleated cells produced by megakaryocytes through megakaryopoiesis (20). They play a critical role in facilitating hemostasis, preventing bleeding, and contributing to the repair of injured blood vessels. The surface coating of platelets can adsorb plasma proteins and clot factor III, whereas platelet granules contain substances associated with blood clotting. When blood vessels are injured or ruptured, platelets are stimulated and transition from a quiescent state to an active state. Deformation occurs immediately, and subsequently, platelets coagulate into clusters with an increase in surface viscosity. Simultaneously, under the influence of surface III factors, prothrombins in the plasma are converted to thrombi, which catalyze fibrinogen to form fibrin filaments that combine with blood cells to initiate clot formation and stop bleeding. The release of platelet-derived microparticles promotes hemostasis and coagulation (21, 22). Beyond their role in hemostasis, platelets regulate vascular tone, mediate inflammatory responses, participate in host defense mechanisms, and modulate tumor biology (23). Any deviation in the quality and quantity of platelets may result in pathological conditions, including hemorrhagic manifestations due to thrombocytopenia or platelet dysfunction, as well as thrombotic events associated with thrombocytosis.

However, limited data are available regarding the relationship between phthalates and hematological parameters. Arrigo et al. (24) found that phthalates can penetrate erythrocytes and combine with hemoglobin, inducing structural and functional alterations that contribute to anemia. Previous studies on phthalates and platelets revealed a dose-response relationship between phthalate exposure and platelet microparticle levels in adolescents (25), and phthalate exposure has shown the potential to activate platelet function (26). Notably, none of these studies have focused on the relationship between multiple urinary metabolites derived from phthalates and platelet count. This study aimed to examine the correlation between five phthalate metabolites in urine and platelet count, as well as the impact of phthalate exposure on platelet count in adults.

## 2 Methods

### 2.1 Data source and study population

This cross-sectional study utilized data from the National Health and Nutrition Examination Survey spanning 2005 to 2018, focusing on adult participants. Approval of this study was obtained from the ethics review board of the National Center for Health Statistics (NCHS) to assess non-institutional U.S. civilians' health and nutrition status. All participants gave written informed consent. The experimental protocol was established according to the ethical guidelines of the Declaration of Helsinki. Additional details are available on the NHANES official website.

During the study periods, 70,190 individuals were enrolled, including 39,041 non-pregnant participants aged 20 years or older. Individuals with missing platelet data (*n* = 3424) and phthalate metabolite information (*n* = 24,208) were excluded. Finally, 11,409 participants were included in this study.

### 2.2 Phthalate metabolites and platelets assessment

The NHANES team analyzed one-third of the participants in each cycle. Quantitative analysis of phthalate metabolites in urine was performed using high-performance liquid chromatography-electrospray ionization tandem mass spectrometry (HPLC-ESI-MS/MS). Urine samples are processed using enzymatic deconjugation of the glucuronidated metabolites followed by on-line solid phase extraction coupled with reversed phase HPLC-ESI-MS/MS. To enhance assay accuracy, isotopically-labeled internal standards for phthalate metabolites and mono-(hydroxy-n-cyclohexyl) phthalate are integrated into the process. The detailed laboratory procedures are available on the NHANES official website. Compounds with detection rates below 80% were excluded from subsequent analyses. Finally, we focused on five specific phthalate metabolites: MCNP, MCOP, MCPP, MiBP, and MNP. The count of platelet was assessed using a Beckman Coulter Counter (Beckman Coulter Inc., Brea, CA, USA).

### 2.3 Covariates

The covariates included in the model were selected based on previous empirical data regarding the relationship between phthalate exposure and other blood parameters. The categorical covariates encompassed variables, such as age, sex, ethnicity, education, smoking status, alcohol consumption status, body mass index (BMI), and household poverty-income ratio (PIR). Continuous covariates included urinary creatinine levels (mg/dl). In this study, using more than 100 cigarettes in a lifetime was defined as a smoker, and using 12 ounces of beer, 5 ounces of wine, or one and a half ounces in a year was defined as a drinker.

### 2.4 Statistical analysis

Statistical analyses were conducted using R 4.3.1 software. Considering the complexity of the sampling design, survey weights of the NHANES data were taken into account in the statistical analyses. Before modeling analysis, the urinary phthalate metabolite data were normalized by log2 conversion. Continuous variables are presented as means and standard deviations, whereas categorical variables are presented as numerical values and percentages.

A weighted multivariate logistic regression model was used to calculate the odds ratios (OR) and their corresponding 95% confidence intervals (CI) to estimate the impact of a single phthalate metabolite on platelet count. Subsequently, subgroup analyses were performed to assess the association between phthalate metabolite concentration and platelet count to determine whether any disparities existed among the sex and age cohorts. To investigate the correlation between phthalate metabolite level and platelet count, we established a restricted cubic spline (RCS) analysis in a comprehensive model that accounted for all relevant variables. To further investigate potential non-linear dose-response relationships and synergistic interactions among chemical mixtures, we utilized weighted quantile sum (WQS) to evaluate the five phthalate metabolites combinations' combined effects on platelets.

## 3 Results

### 3.1 Characteristics of selected participants

Table 1 summarizes the general characteristics and covariate distributions of the study population. The median age of the participants was 49.7 years (SD = 17.7), and 5,757 (50.5%) were female. 2,855 (25%) were Mexican Americans or other Hispanic, 4,807 (42.1%) were non-Hispanic white, 4,807 (42.1%) were non-Hispanic black, and 1,300 (11.4%) were of different races. 2,823 (24.8%) were below high school, 2,823 (24.8%) had graduated from high school, and 5,943 (52.1%) were above high school level.

**Table 1:** Baseline characteristics of participants in NHANES 2005–2018 cycles.

**Characteristic**	**Total (*n* = 11,409)**
**Age**, ***N*** **(%)**	
20–39	3,764 (33.0%)
40–59	3,825 (33.5%)
≥60	3,820 (33.5%)
**Gender**, ***N*** **(%)**	
Male	5,652 (49.5%)
Female	5,757 (50.5%)
**Race**, ***N*** **(%)**	
Mexican American	1,757 (15.4%)
Other Hispanic	1,098 (9.6%)
Non-Hispanic white	4,807 (42.1%)
Non-Hispanic black	2,448 (21.5%)
Other race	1,300 (11.4%)
**Educational level**, ***N*** **(%)**	
Lower than high school	2,823 (24.8%)
High school	2,629 (23.0%)
Greater than high school	5,943 (52.1%)
Smoker, *N* (%)	5,066 (44.4%)
Drinker, *N* (%)	6,467 (56.7%)
PIR, *N* (%)	5,066 (44.4%)
BMI (kg/m^2^), M ± SD	27.2 (7.01)
Urinary creatinine (mg/dl), M ± SD	2.53 (1.64)

NHANES, National Health and Nutrition Examination Survey; PIR, poverty income ratio; BMI, body mass index; M, mean; N, numbers of subject; SD, standard deviation.

### 3.2 Correlations between phthalate metabolites and platelet count

The correlations between urine phthalate metabolites and platelet count were examined using weighted multiple logistic regression (Table 2). Model 1 was unadjusted for potential confounders. Model 2 considered sex, age, race, and educational attainment. Model 3 was adjusted for sex, age, race, and education level, as well as smoking status, alcohol consumption status, BMI, PIR, and urinary creatinine. We observed a negative correlation between phthalate metabolite level and platelet count. MCOP [OR (95% CI) = 0.032 (0.010–0.100)] was significantly associated with platelet count in Model 1. After adjusting for sex, age, race, and education level, MCOP [OR (95% CI) = 0.037 (0.013–0.110)] still showed a significant relationship with platelet count. These findings remained statistically significant after accommodating for the remaining covariates in Model 3 [OR (95% CI) = 0.009 (0.002–0.036)].

**Table 2:** Correlations of phthalate metabolites with platelet count.

**Phthalate metabolites (ng/ml)**	**Model 1**		**Model 2**		**Model 3**	
	**OR (95% CI)**	***P* value**	**OR (95% CI)**	***P* value**	**OR (95% CI)**	***P* value**
MCNP	0.368 (0.098–1.387)	0.138	0.817 (0.228–2.920)	0.753	0.348 (0.081–1.497)	0.154
MCOP	0.032 (0.010–0.100)	< 0.001	0.037 (0.013–0.110)	< 0.001	0.009 (0.002–0.036)	< 0.001
MCPP	0.733 (0.197–2.736)	0.641	1.169 (0.319–4.281)	0.812	1.460 (0.280–7.617)	0.650
MiBP	1.222 (0.243–6.153)	0.806	0.712 (0.157–3.233)	0.657	0.258 (0.032–2.094)	0.202
MNP	1.269 (0.244–6.594)	0.775	1.054 (0.206–5.397)	0.950	1.597 (0.273–9.348)	0.600

MCNP, mono-(carboxyisononyl) phthalate; MCOP, mono-(carboxyisoctyl) phthalate; MCPP, mono-(3-carboxypropyl) phthalate; MiBP, mono-isobutyl phthalate; MNP, mono-isononyl phthalate. Model 1 was adjusted for none; Model 2 was adjusted for gender, age, race, and educational attainment; Model 3 was adjusted for age, sex, race, education levels, smoking status, alcohol intake, BMI, PIR, and urine creatinine.

### 3.3 Subgroup analysis

The study population was categorized into subgroups based on age and sex. Covariates included race, education level, smoking status, alcohol consumption status, BMI, PIR, and urinary creatinine. By weighted multiple logistic regression analysis, MCOP displayed substantial negative correlations with platelet count across all age and sex groups (Table 3). Among age subgroups, the association between MCOP and platelet count was strongest in people aged 40–59 years [OR (95% CI) = 0.003(0.000–0.019)]. In gender subgroups, the association was more significant in the female population [OR (95% CI) = 0.004 (0.001–0.033)]. Additionally, MCNP was adversely correlated with platelet count in females [OR (95% CI) = 0.083(0.013–0.552)].

**Table 3:** Subgroup analysis of phthalate metabolites with platelet count.

**Subgroup**	**MCNP**	**MCOP**	**MCPP**	**MiBP**	**MNP**
**Age**					
20–39	0.408 (0.043–3.837)	0.028 (0.004–0.212)^***^	0.655 (0.074–5.819)	0.631 (0.041–9.725)	0.650 (0.055–7.655)
40–59	0.119 (0.006–2.280)	0.003 (0.000–0.019)^***^	0.906 (0.050–16.354)	0.058 (0.002–1.525)	3.063 (0.151–62.190)
≥60	3.316 (0.152–72.466)	0.021 (0.002–0.280)^**^	20.619 (0.990–429.248)	0.369 (0.013–10.871)	5.613 (0.151–208.533)
**Gender**					
Male	1.308 (0.129–13.301)	0.015 (0.002–0.093)^***^	0.699 (0.115–4.272)	0.298 (0.020–4.455)	0.380 (0.048–3.001)
Female	0.083 (0.013–0.552)^*^	0.004 (0.001–0.033)^***^	2.846 (0.234–34.542)	0.123 (0.008–1.937)	9.492 (0.31–290.222)

MCNP, mono-(carboxyisononyl) phthalate; MCOP, mono-(carboxyisoctyl) phthalate; MCPP, mono-(3-carboxypropyl) phthalate; MiBP, mono-isobutyl phthalate; MNP, mono-isononyl phthalate. Models were adjusted for age, sex, race, education levels, smoking status, alcohol intake, BMI, PIR, and urine creatinine. ^*^ < 0.05; ^**^ < 0.01; ^***^ < 0.001.

### 3.4 Restricted cubic spline

**Figure 1:**
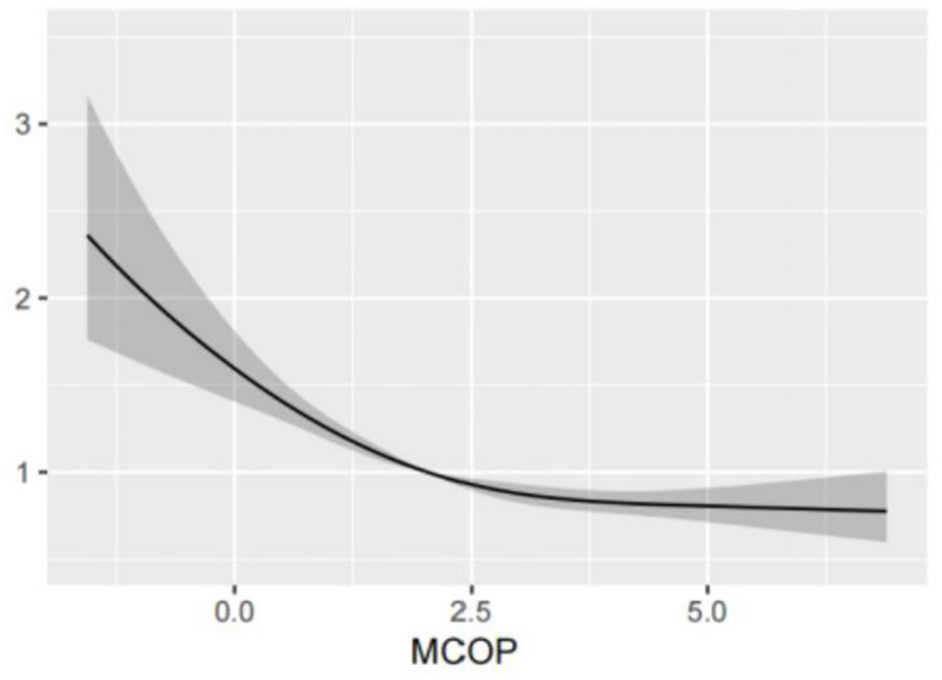
Analysis of restricted cubic spline regression of MCOP for platelet count. MCOP, mono-(carboxyisoctyl) phthalate. The RCS model was adjusted for age, sex, race, education levels, smoking status, alcohol intake, BMI, PIR, and urine creatinine.

Based on the above analysis, there was a strong correlation between the MCOP and platelet count. Therefore, a restricted cubic spline was used for further studies. After adjusting for covariates, including age, sex, ethnicity, education, smoking status, alcohol consumption status, BMI, PIR, and urinary creatinine, the findings revealed a statistically significant nonlinear relationship between MCOP and platelet count (*P* < 0.001). Details are shown in Figure 1.

### 3.5 Weighted quantile sum

**Figure 2:**
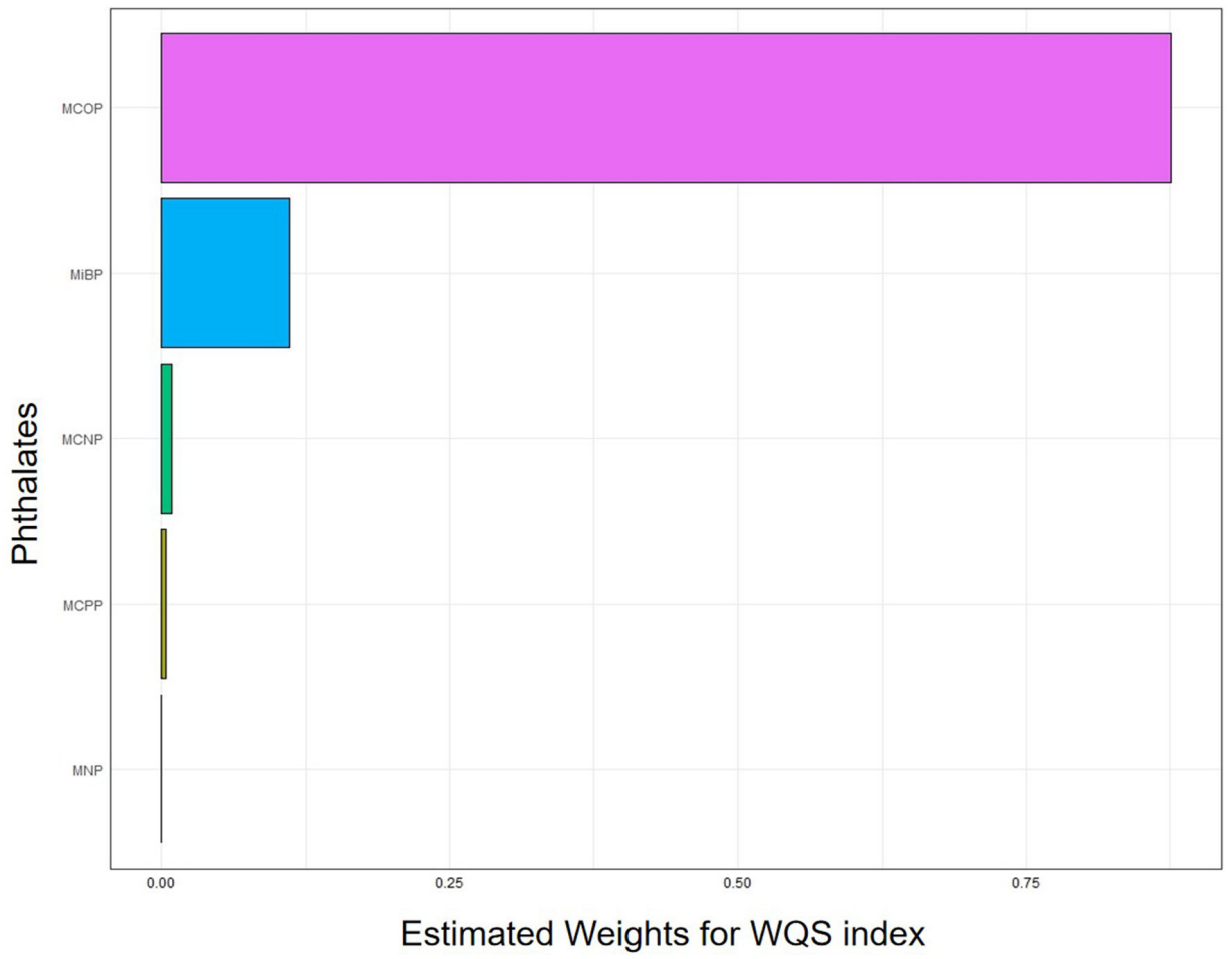
Analysis of weighted quantile sum regression of MCOP for platelet count. MCOP, mono-(carboxyisoctyl) phthalate; MiBP, mono-isobutyl phthalate; MCNP, metabolites, mono-(carboxyisononyl) phthalate; MCPP, mono-(3-carboxypropyl) phthalate; MNP, mono-isononyl phthalate. The WQS model was adjusted for age, sex, race, education levels, smoking status, alcohol intake, BMI, PIR, and urine creatinine.

Taking age, sex, ethnicity, education, smoking status, alcohol consumption status, BMI, PIR, and urinary creatinine as covariables, the WQS results showed that the MCOP accounted for the highest proportion of platelet count in the negative role. Details are presented in Figure 2 and Table 4.

**Table 4:** Weight of different phthalate metabolites influence on platelet count in WQS model.

**Phthalate metabolites**	**Weight**
MCOP	0.88
MiBP	0.11
MCNP	0.01
MCPP	0.00
MNP	0.00

MCOP, mono-(carboxyisoctyl) phthalate; MiBP, mono-isobutyl phthalate; MCNP, metabolites, mono-(carboxyisononyl) phthalate; MCPP, mono-(3-carboxypropyl) phthalate; MNP, mono-isononyl phthalate. The WQS model was adjusted for age, sex, race, education levels, smoking status, alcohol intake, BMI, PIR, and urine creatinine.

## 4 Discussion

This cross-sectional study presented evidence indicating the influence of phthalate metabolites on platelet count in adults in the United States, which included data from the NHANES between 2005 and 2018. To the best of our knowledge, only a few studies have explored the impact of phthalate metabolites on platelet count, and no association has been found (27, 28). Our results showed a significant inverse relationship between MCOP and platelet count. To analyze this relationship in more detail, we performed a subgroup analysis. The analysis found that the negative association between MCOP and platelet count was more pronounced in female and in people aged 40–59 years. This finding further strengthens the idea that MCOP affects platelet count in specific populations. In addition, we performed further analyses using RCS, which showed a significant nonlinear correlation between MCOP and platelet count. This finding not only reveals the complexity of the relationship between MCOP and platelet count, but also provides new ideas for follow-up research. In subgroup analyses, we also found that MCNP was inversely associated with platelet count in female. This finding further expands our understanding of the relationship between phthalate exposure and platelet count and suggests that we need to pay more attention to the effects of different metabolites in different populations in the future.

Platelet generation is a sophisticated process that encompasses the regulation of hematopoietic stem cells (HSCs) and their differentiated progeny, bone marrow microenvironment, and hematopoietic cytokines (29). Phthalates and its metabolites may participate in the maintenance of the platelet count by affecting one or more of these links.

Short-term exposure to BBP decreases the number of bone marrow cells in adult male rats, suggesting that BBP has adverse effects on the hematopoietic system (30). The maturation of megakaryocytes and the formation of platelets rely on the migration of cells from osteoblasts to vascular niches (31). Osteoblasts secrete thrombopoietin and support the survival and retention of stem cells, while thrombopoietin promotes DNA repair in HSCs (32). However, experiments *in vitro* have suggested that exposure to DEHP and its metabolite mono(2-ethylhexyl)phthalate (MEHP) may inhibit osteoblast formation through Wnt/β-catenin regulation (33).

Megakaryogenesis is driven by thrombopoietin (TPO), which is constitutively expressed in the liver, kidneys, bone marrow, and smooth muscle (34). Hepatocytes capture desialylated platelets via AMR and activate the JAK2-STAT3 signaling pathway to induce the synthesis of TPO (35). However, phthalates can modify the structure and functional integrity of the liver by inducing alterations in peroxisomes, mitochondria, and enzymes associated with fatty acid transport and beta-oxidation (36). Furthermore, thrombocytosis promotes hepatocyte mitosis, whereas a reduction in platelet count results in impaired liver regeneration (37–40). Murone et al. showed that TPO-deficient mice exhibit a 90 percent reduction in platelet count (41). Di-2-ethylhexyl adipate (DEHA), a common alternative to DEHP, has also been linked to the development of liver tumors in male mice (42), and it needs to be further verified whether it has an effect on the production of TPO. TPO can also be produced by the kidney; however, its function and regulatory mechanisms still need to be determined (43, 44). The renal toxicity of phthalate metabolites may also affect the production of TPO (45).

In addition, there is an inverse correlation between urinary phthalate metabolites and total thyroxine (T4), and MCOP has the most significant impact on the mixing effect (46, 47). Serum T4 levels are independently associated with platelet count, but the underlying biological mechanism remains poorly understood (48). The number of megakaryocytes in bone marrow aspirates from patients with hyperthyroidism is significantly higher than that in healthy individuals (49). Thyroid hormones may affect platelet production, prolong platelet survival, and enhance megakaryocyte proliferation by modulating bone marrow matrix proteins, such as fibronectin (48). Moreover, thyroid hormones upregulate the expression of fibronectin in specific cell lines, which correlates with elevated fibronectin levels in hyperthyroidism patients (50, 51). Notably, fibronectin appeared to affect the maturation of megakaryocytes via its interaction with integrin α4β1 (52). In parallel, while apoptosis is the main mechanism of platelet death, thyroid hormones demonstrate anti-apoptotic effects in multiple cell lines (53).

Furthermore, studies have demonstrated that exposure to phthalates may result in decreased androgen levels (54). Conversely, physiological doses of androgen have been found to increase platelet synthesis and accelerate platelet activity (55). Supporting this mechanism, castrated BALB/c mice exhibited diminished platelet production, whereas maintenance doses of testosterone can restore platelet production to normal levels within a few days. These findings suggest that testosterone may exert its effects on bipotential hematopoietic precursor cells (56).

However, the effects of phthalates on platelet count are complex and uncertain. Several cross-sectional investigations have demonstrated the connection between phthalates and the upregulation of inflammatory factors such as leukocytes, C-reactive protein, TNF-alpha (TNF-α), and interleukin-6 (IL-6) (57–59). Nevertheless, the increase in thrombopoietin expression induced by inflammation is mediated by IL-6, which increases thrombopoietin production in hepatocytes both *in vivo* and *in vitro* (60). Trim et al. (61) measured 13 kinds of phthalate metabolites in the urine of menopausal women. Among these, MCNP was the sole metabolite showing a positive correlation with IL-6 level [β = 0.108; 95% CI (0.013, 0.204)], whereas no significant correlations were observed between the remaining 12 metabolites and IL-6 level. In addition, TNF-α not only activates immune cells, such as macrophages and T lymphocytes, to promote the immune-mediated destruction of platelets but also can activate signaling pathways, such as MAPK-ERK1/2, to inhibit platelet production (25, 26).

We conducted the first investigation into the relationship between phthalate metabolite and platelet count in the United States, utilizing a sufficiently large sample size for representativeness. Several statistical models have been employed to examine the individual and combined effects of phthalate metabolites on platelet count, producing generally consistent results.

This study has several limitations. First, the current study was based on a cross-sectional study design, so further longitudinal and experimental studies are needed to establish a definitive cause-and-effect relationship. Second, phthalate metabolites exhibit a short half-life in urine, and do not necessarily reflect the effects of long-term exposure. Third, other residual or unmeasured confounders may have influenced the results after accounting for and controlling for significant confounders, such as the complexity of phthalate metabolism in humans and co-exposures to other environmental pollutants. Finally, participants with hematological disorders were not considered invalid. Therefore, more prospective cohort studies are necessary to investigate and confirm the association between phthalate metabolite and platelet count.

## 5 Conclusion

In conclusion, higher urinary MCOP level was associated with lower platelet count. A longitudinal and experimental study investigating the biological mechanisms underlying the effects of phthalate metabolites on platelets is warranted.

## Data Availability

The datasets presented in this study can be found in online repositories. The names of the repository/repositories and accession number(s) can be found below: https://wwwn.cdc.gov/nchs/nhanes/Default.aspx.
